# Sponges, ctenophores, and the statistical significance of syntenies

**DOI:** 10.1093/molbev/msaf321

**Published:** 2025-12-08

**Authors:** Richard R Copley

**Affiliations:** Laboratoire de Biologie du Développement de Villefranche-sur-mer, Institut de la Mer de Villefranche-sur-mer, Sorbonne Université, CNRS UMR7009, Villefranche-sur-mer 06230, France

## Abstract

Shared fusions between ancestral chromosomal linkage groups have previously been used to support phylogenetic groupings, notably sponges with cnidarians and bilaterians to the exclusion of ctenophores, rendering ctenophores the sister group to all other animals. The linkage groups used to identify these fusions were assessed for statistical significance relative to a model of randomly shuffled genes. I argue that the method of random shuffling treated all species as equally distant from each other and so overestimated the significance of the observed linkages. I calculate alternative statistics and further argue that there are likely to be real linkage groups that are not identified as significant. If linkage groups are not supported statistically, they cannot reliably be used to identify shared derived chromosomal rearrangements, and hence phylogenetic hypotheses derived from them are suspect.

## Introduction

Whether ctenophores or sponges are sister group to the other animals has been the subject of continuing dispute since the first “phylogenomic” era study to include ctenophores (Dunn et al. 2008). In that work, Dunn et al. assembled a multigene, multispecies sequence alignment and found several surprising phylogenetic relationships, among which, ctenophores as the earliest branching animal phylum attracted much interest. Since then, there has been some degree of back and forth between camps purporting to show ctenophores or sponges in this position (Pisani et al. 2015, 2016; Halanych et al. 2016; Feuda et al. 2017; Simion et al. 2017; Whelan et al. 2017; Kapli and Telford 2020; Szánthó et al. 2023; Liu et al. 2024; Steenwyk and King 2025). Very broadly speaking, analyses focused on complex phylogenetic models have tended to show sponges sister, whereas those focused on assembling large data sets, ctenophores (but see Li et al. 2021). Other sources of data besides sequence alignments have been explored (eg gene content (Leclère et al. 2019; Pett et al. 2019)), but these results have not had wide influence. Gene synteny data have recently been proposed as a possible independent source of informative phylogenetic markers. Schultz et al. (2023) (henceforth SSR23) used inferred ancestral linkage groups (ALGs) to hypothesize a number of polarized chromosome fusion events that were uniquely shared between sponges and the clade of cnidarians and bilaterians (ie parahoxozoans), thus supporting ctenophore sister to other animals. SSR23 tested the statistical significance of the initial inferred ALGs through random permutation of chromosomal gene order. I suggest that the main procedure they adopted leads to inflated estimates of significance and that when more appropriate tests are used, support for the conservation of some ALGs disappears or is sensitive to the use of closely related genomes or the method of orthology identification.

SSR23 defined synteny groups with respect to four species quartets: a unicellular outgroup, a sponge, a ctenophore, and a cnidarian or bilaterian. The number of orthologs, *N*, shared by potential synteny groups was assessed for significance by randomly permuting the gene order across all chromosomes within every species of the quartet and counting the number of times a group of *N* orthologs was observed. These values were used as estimates of the false discovery rate (FDR). There are two problems with this procedure. Firstly, by permuting all species, it takes no account of the fact that the species show greatly differing levels of phylogenetic relatedness. To see that this is a problem, imagine the hypothetical case of adding another species whose synteny is perfectly conserved with one already in the dataset. To correctly simulate the biological reality, the chromosomes of these identical species should be permuted in exactly the same way—they should not be treated as statistically independent. Instead, if all chromosomes are randomly permuted, purely by adding the new species, the statistical significance of any synteny group in the unpermuted data will be lowered (ie made more significant) because random failure becomes more likely, despite the underlying evolutionary history remaining the same.

The second problem with the procedure of SSR23 is the synthesis of the permuted data into a set of FDRs for a given gene group size (eg five orthologs have an FDR of *x*). These values give a probability for a group size averaged across all chromosomes. We might reasonably expect, however, that the statistical significance should depend on the number of shared genes relative to the sizes of the chromosomes that the linkages are observed on. A group of five genes shared on small chromosomes of the four species is less likely than the same group size shared by the largest chromosomes.

While the first of these problems, that of permutation of all species, will lead to more erroneously significant results, the effect of the second is less clear a priori as it will depend upon which chromosomes are likely syntenic. To investigate this further, I implemented an alternative approach that addresses both issues.

## Results

### Shuffling all species inflates statistical significance

**Figure 1:**
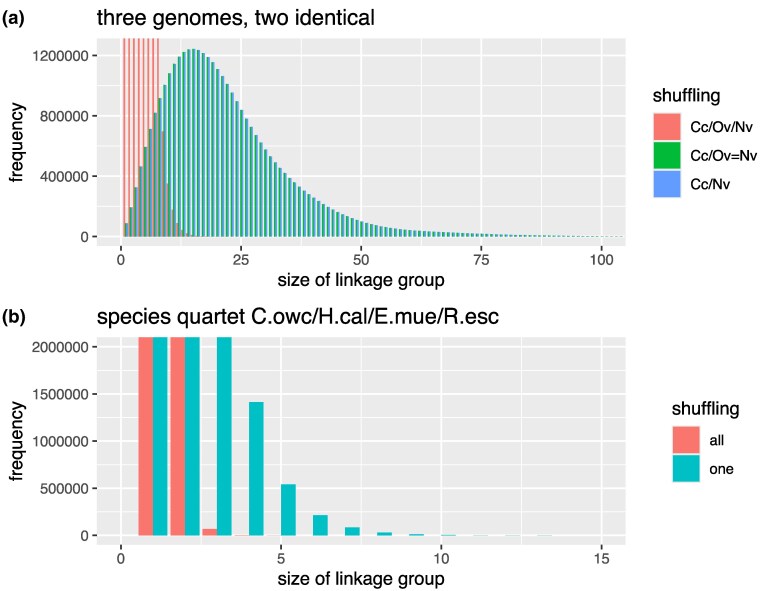
Effects of shuffling just one species vs. all species. The bar plot bins show the frequency of a given number of linked orthologs for 100,000 sets of randomized gene location data. In a) the blue bars illustrate a pair of species, with one shuffled *C.can/N.vec*). The green and red bars illustrate a trio of species where two are identical (*C.can/O.vec* = *N.vec*). In the green series, only the locations of *C.can* genes were shuffled. In the red series, the locations of all genes were shuffled. The desired biological behavior, an identical distribution to a pair of species, is seen with the shuffle one strategy. b) Shufflings based on *C.owc*, *H.cal*, *E.mue*, and *R. esc* ortholog groups. In the blue bars, only *C.owc* ortholog locations have been shuffled. In the red bar series, all species ortholog locations were shuffled.

In order to illustrate the effects of different permutation strategies, I developed a code to calculate the number of times a particular combination of chromosomes was supported by shared orthologs. (That within all species, all gene locations across all chromosomes are shuffled in SSR23 permutation tests is not completely obvious from the method text, which refers to “10 million permutations of gene indices” (supplementary information, p.58 (Schultz et al. 2023)), but it is apparent from the archived code odp_nway_rbh (https://zenodo.org/records/7857390 lines 415–6), where each thisscaf is a dataframe column representing a species.) Using the data of SSR23, this exactly reproduced the sizes of the SSR23 shared ortholog groups. I then made a dataset of orthologs with chromosomal locations for the cnidarian *Nematostella vectensis* and the sponge *Corticium candelabrum*, adding a fake third species by copying the *Nematostella* gene locations to a set of new chromosome names and made-up gene ids, which had a one-to-one mapping to the *Nematostella* data. To calculate the random distribution of chromosome combinations, I repeatedly shuffled the gene to chromosome mapping for a given species. I either permuted just the *Corticium* gene locations (ie the outgroup) relative to the two identical species or the locations of all species (Fig. 1a). When all species are shuffled, the frequency with which random chromosomal groups are supported by larger numbers of genes is greatly reduced, which would make observed “real” groupings more significant. When the data of SSR23 (Schultz et al. 2023) are used for the quartet of *Capsaspora owczarzaki*, *Hormiphora californensis*, *Ephydatia muelleri*, and *Rhopilema esculentum*, shuffling all species gives numbers comparable to those found in SSR23: groups of five linked orthologs are seen extremely rarely and groups of eight never seen. In contrast, shuffling just the *C.owc* ortholog locations yields larger linked groups (Fig. 1b).

### The outgroup comparison is the hard to pass test

The example with closely related genomes above suggests that the strategy of shuffling all gene to chromosome assignments has undesirable behavior, reducing the data to totally independent observations even when there is underlying evolutionary structure.

Given the pairwise Oxford dot plots of ctenophore, sponge, and cnidarian syntenies, it is apparent that the gene locations of representatives of these species are correlated (Schultz et al. 2023) and so likely affected by this problem. Further inspection of dot plots and the statistical significance of pairwise comparisons (Simakov et al. 2022; Schultz et al. 2023) suggests that the critical test for whether data can be used to address the phylogenetic position of sponges or ctenophores is that of the significance of metazoan/unicellular outgroup combinations (compare SSR23 Extended Data Figs. 2 and 7). One way of looking at this problem is to consider each of the metazoan chromosome groups linked by shared orthologs as a hypothesis of an ancestral metazoan chromosome. We need not worry about the validity of the metazoan grouping in itself at this stage, as if it is not real, it will not share orthologs with unicellular chromosomes in anything other than a random manner. Each of the observed metazoan groupings can be tested as a unit against the chromosomes of a unicellular genome, to determine if there is a significant enrichment of shared genes—if we randomly scattered all the unicellular orthologs of the metazoan grouping across the unicellular species chromosomes, how likely would we be to see the number we do see, or a larger number, by chance on our chromosome of interest? This probability under a null model (no significant enrichment) can be determined using the hypergeometric distribution (effectively Fisher's exact test). For example in SSR23, the ctenophore/sponge/cnidarian chromosome group HCA7, EMU19, and RES2 shares 15 orthologs with *Capsaspora* COW3. We can sum the total number of orthologs shared by the HCA7, EMU19, and RES2 combination, with any *Capsaspora* chromosome, giving 27, and the number of orthologs on COW3 as 182. We also know the total number of orthologs in our data (giving ((15,167),(12,1680)) as a contingency table). Accordingly, the uncorrected *P*-value for this comparison, ie the probability of observing 15 or more orthologs on these chromosomes, is, from the survival function of the hypergeometric distribution, 2.3e−09. Using this approach, we can calculate bespoke *P*-values for each hypothesis of metazoan–unicellular chromosome orthology without relying on a general threshold that does not consider the overall number of orthologs on a chromosome (which serves as a proxy for size). A multiple testing correction can then be applied, as each combination of chromosomes counts as a hypothesis tested. These values are compared to those in SSR23 in Table 1.

**Table 1:** Recalculated significance of linkage groups for *S.ros* (SRO) and *C.owc* (COW) with *H.cal*, *E.mue* and *R.esc* (HCA, EMU, RES) modified from SSR23.

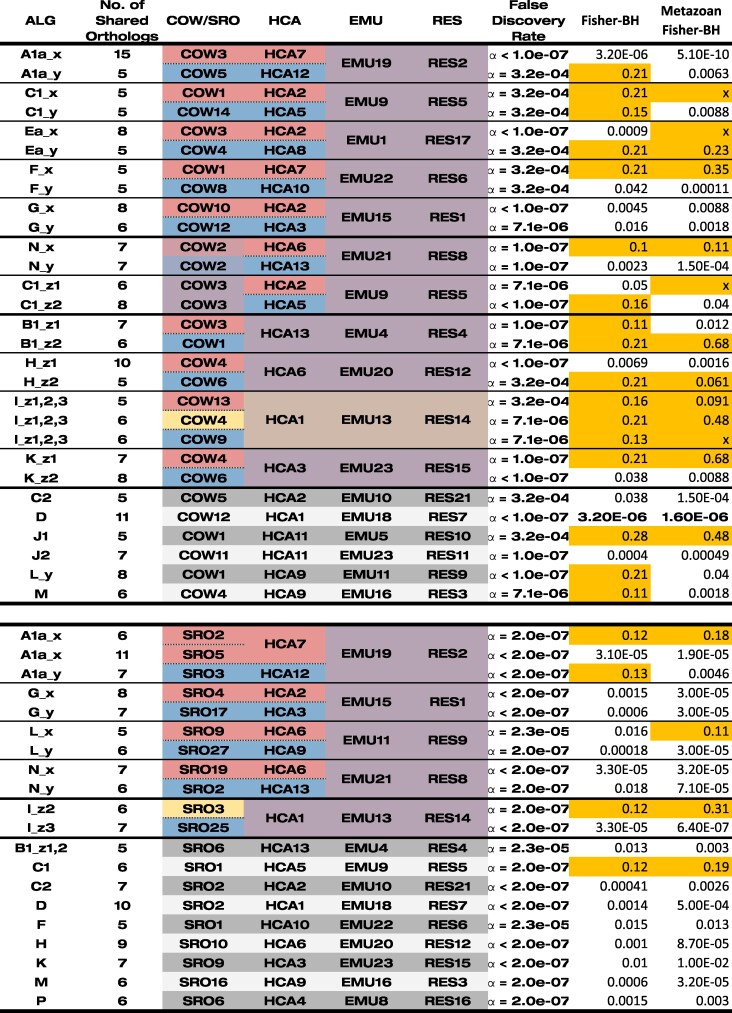

The “False discovery rate” gives the SSR23 statistics. The column “Fisher-BH” uses the orthology groups of SSR23 and gives Benjamini-Hochberg–corrected *P*-values using the procedure described in the text. The column “Metazoan Fisher-BH” uses recalculated orthology groups and first identifies likely ancestral metazoan chromosomal combinations to reduce the number of tests, as described in the text. Where an “x” is placed in this column, the metazoan chromosomal combination was not supported.

**Figure 2:**
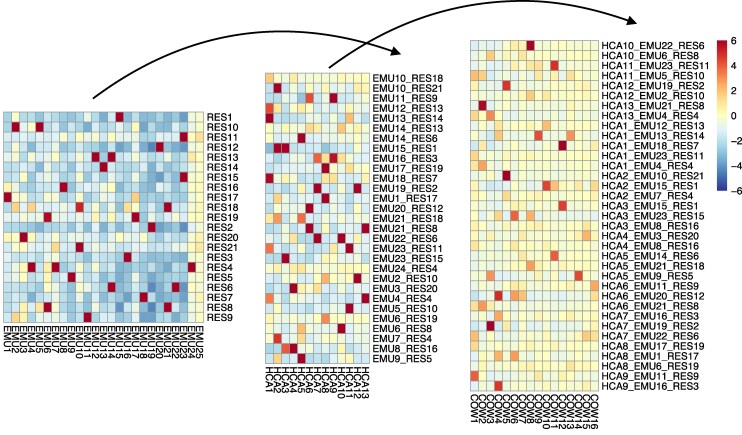
Example of the hierarchical buildup of unicellular–metazoan syntenies. Each grid shows a heatmap representing counts of shared orthologs for all vs. all chromosome (or combinations of chromosome) comparison, with all individual cells colored, on the same scale, by the standardized Pearson residuals for the omnibus chi-squared test represented in the heatmap. Significant syntenies between *Rhopilema* and *Ephydatia* (left) (the more red squares) are used as the basis for tests against *Hormiphora* (middle). The resulting syntenies are then compared to *Capsaspora* (right). Note that the distinction between true positives and noise becomes less clear across the comparisons, which is likely to lead to more acceptance of false positives and/or rejection of true positives.

We can reduce the number of statistical tests that need to be corrected for by determining which metazoan chromosome combinations are likely to harbor ALGs, so that we only compare these composites to the chromosomes of the unicellular outgroup. I implemented a procedure to build up possible ancestral chromosomes for which there was strong statistical support, comparing first *Rhopilema* (cnidarian) and *Ephydatia* (sponge) and then the results of that analysis with *Hormiphora* (ctenophore). These chromosomal combinations were then compared to the outgroup. This lets us make use of the greater number of orthologs found in the pairwise comparison between *Ephydatia* and *Rhopilema* and then within the metazoans when there is no unicellular outgroup and so should a priori be favored (see Materials and methods and Fig. 2). The results are shown in the column “Metazoan Fisher-BH” in Table 1.

### The minor agreements

In *Capsaspora*, the genes XP_004365359.1 and XP_004365360.2 are at adjacent chromosomal locations, and orthologs are found as adjacent pairs in *Ephydatia*, *Rhopilema*, and Bilateria. They are not adjacent in *Hormiphora* but are on the same chromosome, giving a combination of COW1, HCA1, EMU20, and RES11. A similar situation is found with XP_004344437.1 and XP_004344438.2 orthologs on COW12, HCA8, EMU18, and RES7. These combinations of chromosomes are not reported in SSR23 or detected as significant by the analyses above, but we can be sure that the gene pairs represent ALGs because of the extreme unlikelihood of the adjacent gene pairing being found in all species for any reason other than by descent. If convergence driven by function is conjectured, then it is unclear why that should not also be possible with the examples of SSR23. Whereas it might be argued that the case of moving a small number of genes is not the same as a potential chromosome fusion or fission, that is also true for the ALGs of SSR23, which are based on numbers that are not dissimilar relative to the numbers of genes on a chromosome.

## Discussion

I have argued here that SSR23 has overestimated the significance of the linkage groups that they have identified, owing to an overly aggressive permutation strategy. Where SSR23 use more conservative shuffling, for example in their Extended Data Fig. S10, they still compare to metrics based on “real” linkage groups that they have identified as significant with their original criteria. The fact that, in that analysis, even *shuffled* data recover ctenophore sister but never sponges (Schultz et al. 2023) seems problematic for a null model, indicating that some of the ctenophore sister signal within the unshuffled results might be intrinsic to the nongene–order relationships in the data (for instance, number of chromosomes or reliability of ortholog identification) and indicating some degree of long branch effect in the unshuffled results.

Although several SSR23 synteny groups are confirmed in my analysis, their central phylogenetic argument relies on fusions of synteny groups that can be used as synapomorphic phylogenetic markers—thus, both synteny groups involved in a proposed fusion must be statistically significant. With the *Capsaspora* outgroup, using my preferred hierarchical scoring scheme, I recover the synteny groups of the fusions of A1a_x with A1a_y and G_x with G_y. With *Salpingoeca*, I recover these same groups and additionally N_x with N_y. Thus, I contend that three potential fusions should be removed from the SSR23 *Capsaspora* fusion data and one from the *Salpingoeca*. It is worth noting that the A1a and G examples are the only ones that are conserved between outgroups in the analysis of SSR23, but neither represent examples of fusion with mixing. In neither case is there substantial mixing in the sponge. This should be judged within the context of the case of ALG N, where SSR23 treat the lack of full mixing as an argument for convergent fusion in *Capsaspora* (SSR23, supplementary information, p.60). A further complication is that A1a_x and A1a_y are on “predominantly separate” chromosomes in glass sponges and “retain the ancestral state” (SSR23 Extended Data Fig. 3 legend). If this is interpreted as a likely reversion, it nonetheless also calls into question the power of these events as robust phylogenetic markers, not prone to homoplasy. A final issue is that although fusion with mixing may be irreversible, it may not be possible to identify convergent fusion with mixing events unless the true phylogenetic relationships are otherwise evident (see, eg. ALGs B2 and J1, convergently fused with mixing in the annelids *Owenia fusiformis* and *Paraescarpia echinospica* (Fig. 5 in Hilali et al. 2025).

Whether or not they are nominally significant, there is no way of experimentally testing whether syntenies supported by small numbers of genes are syntenic by descent or convergence. The reanalysis presented here shows “ALGs” that, as far as comparisons with the unicellular outgroups go, look, at least in part, as if they were drawn from the right tail of a random probability distribution. It is reasonable to conclude from Fig. 2 that the overlap between signal and noise increases with phylogenetic distance. Given this blurring of real syntenies and noise, it must be further remembered that absence of statistically supported evidence cannot be used to argue that further ALGs sharing few genes do not exist. Rather, from an examination of conserved adjacent pairs of genes, it seems that they do. We do not know how much these might affect the arguments of SSR23. A further cautionary point is that a fully permuted genome is likely to represent an exaggerated level of randomness relative to that which is biologically accessible.

In many instances of the use of fused chromosomes to infer phylogenetic relationships, the statistics should be relatively uncontroversial. This would be the case, for instance, when chromosome fusions in different species share dozens or hundreds of genes. But the use of syntenies whose significance is supported by very few genes should be viewed critically, in line with any other sort of phylogenetic evidence.

## Materials and methods

### Sequence data

For *Capsaspora*, *Salpingoeca*, *Hormiphora*, *Ephydatia*, and *Rhopilema*, protein sequence predictions and chromosomal locations were taken from the supplementary information of SSR23. The A form of *Capsaspora* was used, noting SSR23's observation that this choice makes no difference to results. Gene order and protein sequences for the homoscleromorph sponge *C. candelabrum* and the anthozoan cnidarian *N. vectensis* were taken from the National Center for Biotechnology Information (accessions GCF_963422355.1 and GCF_932526225.1).

### Ortholog identification

I calculated four-way orthologs using the ssearch3 program, an implementation of the Smith–Waterman algorithm from the FASTA package (Pearson and Lipman 1988). Reciprocal best hits, with an *E*-value of <0.1 for pairwise comparisons, were collected and cross-referenced to produce fully consistent four-way species clusters; ie for each ortholog group, single genes from all four species were each other’s best hits (see GitHub code repository for implementation details). Where stated I used the four-way orthologs provided in the supplementary information of SSR23: COW_EMU_HCA_RESLi_reciprocal_best_hits.rbh and EMU_HCA_RESLi_SRO_reciprocal_best_hits.rbh. Both approaches produce similar results.

### Chromosome comparisons

For all four-way orthologs, an outgroup (the single-celled eukaryote, so either *Capsaspora* or *Salpingoeca*) and an ingroup, namely the metazoans *Hormiphora*, *Ephydatia*, and *Rhopilema*, were defined. For each orthologous group, chromosome ids were retrieved for the four genes. A table of ingroup and outgroup chromosome counts was created, where the three ingroup chromosome ids were combined to give one identifier (eg the three ids HCA7 RES2 EMU19 became the single id HCA7_RES2_EMU19), iterating over all orthologous groups. Then, for each unique combination of four chromosomes with shared orthologs (eg COW3, with HCA7_RES2_EMU19, *N* orthologs), the total number of observations of that combination, the total number of orthologs on COW3, the total number of orthologs on HCA7_RES2_EMU19 combinations, and the total number of four-way orthologs in our dataset were retrieved. An exact probability of seeing *N* or more observations was calculated using the hypergeometric distribution. The set of *P*-values for all pairwise comparisons was then corrected for multiple testing using the Benjamini–Hochberg procedure, with an alpha of 0.05.

This procedure was generalized to allow four-way comparisons to be built up from two-way and three-way comparisons, restricting four-way comparisons to chromosome combinations that had been judged significant in three-way comparisons, and three-way comparisons to those significant in two-way, as illustrated in Fig. 2. The standardized residuals used in this figure were calculated using the standardized_resids method of the Python statsmodels module, but are not used in the main calculations.

### Conserved adjacent pairs of orthologs

Conserved adjacent pairs of orthologs were identified using the approach described in Leclère et al. (2019).

## Data Availability

Code and data underlying this article are available at https://github.com/rcply/synteny.
